# Comprehensive draft of the mouse embryonic fibroblast lysosomal proteome by mass spectrometry based proteomics

**DOI:** 10.1038/s41597-020-0399-5

**Published:** 2020-02-26

**Authors:** Srigayatri Ponnaiyan, Fatema Akter, Jasjot Singh, Dominic Winter

**Affiliations:** 1 Institute for Biochemistry and Molecular Biology, Medical Faculty, University of Bonn, Bonn, 53115 Germany; 2 Department of Pharmacology, Faculty of Veterinary Science, Bangladesh Agricultural University, Mymensingh, 2202 Bangladesh

**Keywords:** Lysosomes, Proteomics, Proteomic analysis

## Abstract

Lysosomes are the main degradative organelles of cells and involved in a variety of processes including the recycling of macromolecules, storage of compounds, and metabolic signaling. Despite an increasing interest in the proteomic analysis of lysosomes, no systematic study of sample preparation protocols for lysosome enriched fractions has been performed to date. In the current study, we used samples enriched for lysosomes by paramagnetic nanoparticles and systematically evaluated experimental parameters for the analysis of the lysosomal proteome. This includes different approaches for the concentration of lysosome-containing fractions; desalting of samples by solid phase extraction; fractionation of peptide samples; and different gradient lengths for LC-MS/MS analyses of unfractionated samples by data dependent and data independent acquisition. Furthermore, we evaluated four different digestion methods including filter aided sample preparation (FASP), in-gel digestion, and in-solution digestion using either RapiGest or urea. Using the combined data, we generated a benchmark lysosomal proteome data set for mouse embryonic fibroblasts as well as a spectral library for the analysis of lysosomes by data independent acquisition.

## Abstract (summary)

**Table:** 

Measurement(s)	Proteome
Technology Type(s)	liquid chromatography-tandem mass spectrometry
Sample Characteristic - Organism	Mus musculus

Machine-accessible metadata file describing the reported data: 10.6084/m9.figshare.11770893

## Background & Summary

Lysosomes are the main degradative compartments of mammalian cells and contain a variety of hydrolases which catalyze the breakdown of virtually any cellular macromolecule. Malfunctions of hydrolases leads to the accumulation of their respective substrate, resulting in so-called lysosomal storage disorders (LSDs), a group of about 50 genetically different but phenotypically connected severe diseases^1^. Due to the direct relation between lysosomal hydrolase malfunction and disease phenotype, this group of enzymes has been thoroughly investigated, and the mechanisms of cellular macromolecule degradation in lysosomes are relatively well understood^2^. While no mechanisms for the regulation of lysosomal hydrolases are known, it is becoming more and more apparent that lysosomes play an important role in the distribution and regulation of cellular metabolites, and that they are significantly involved in cellular signaling, which is regulated e.g. by phoshorylation^3^. Furthermore, it is by now well-established that the impairment of lysosomal function plays often a crucial role in more common diseases as e.g. neurodegenerative disorders^4^ and cancer^5^. Therefore, there is an increasing interest in the analysis of lysosomes.

The method of choice for the unbiased analysis of organelle-specific proteomes is mass spectrometry-based proteomics. Due to the low abundance of lysosomal proteins in mammalian cells, their enrichment is a prerequisite for proteomic analysis. Generally, it can be differentiated if a method aims at analyzing the whole proteome, which is divided in subcellular fractions, and lysosomes are one of them^6^, or if lysosomes are the main target of the analysis^7^.

One of the most commonly used methods for subcellular fractionation is density gradient centrifugation^6,8^. Several discontinuous approaches have been developed for the generation of lysosome enriched fractions using Mitrizamide^9^, Nycodenz^10^, and Percoll^11,12^. To further increase specificity, a change in lysosome density can be induced e.g. by injection of Triton-WR1339 in animals, which leads to a liver-specific change of lysosomal density^13^.

Another commonly used method utilizes the specific targeting of magnetic nanoparticles (iron dextran particles (FeDex^14^)/superparamagnetic iron oxide nanoparticles (SPIONs^15^)) to the lysosomal compartment by delivery through unspecific fluid-phase endocytosis. The particle-containing lysosomes can then be isolated through a magnetic field. This approach has been utilized in several studies for comparative proteomics experiments as well as lipidomics studies^7,16^. Recently, another approach has been introduced for the enrichment of lysosomes, the immunoprecipitation via tagged lysosomal membrane proteins. This approach was initially established using a RFP-Flag tagged version of the lysosomal membrane protein Lamp1^17^, and later extended to a HA-tagged version of TMEM192, which was utilized for the metabolomic and proteomic analysis of isolated lysosomes^18,19^.

Besides the enrichment of lysosomes as an intact organelle, a unique feature of lysosomal matrix proteins has been used extensively for their affinity purification: their posttranslational modification with mannose 6 phosphate (M6P), which acts as a lysosomal targeting signal^20^ and is removed by the acid phosphatases ACP2 and ACP5^21^ in the lysosomal lumen. Lysosomal proteins which are still carrying the M6P residue can be enriched by immobilized domains of the M6P receptors MPR46/MPR300, or resins used for the enrichment of phosphopeptides, such as IMAC^22^. This approach has been applied to mouse embryonic fibroblasts deficient for both MPRs^23^, to human brain and plasma samples^24,25^, and to 17 individual rat tissues^26^, revealing novel proteins of potential lysosomal origin. Additionally, changes in the lysosomal proteome in a mouse model of Niemann Pick Disease Type C^27^ and in patients affected by LSDs of unknown etiology^28^ were investigated. In order to increase the amount of M6P modified proteins, mice and cells deficient for ACP2 and ACP5 were used in several studies^22,29,30^.

**Fig. 1:**
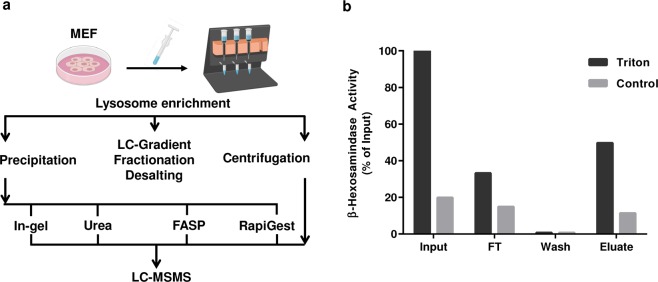
Sample processing workflow and assessment of lysosomal yield. **(a)** Workflow for the sample preparation of MEF lysosome enriched fractions for LC-MS/MS analysis. Individual parts of the protocol which were evaluated are indicated. **(b)** Assessment of the efficiency of lysosome enrichment and lysosomal integrity by enzymatic β-hexosaminidase assays. Differences in enzymatic activity between control (intact lysosomes) and triton treated (disrupted lysosomes) samples allow to determine the amount of intact lysosomes in the individual fractions. MEFs: mouse embryonic fibroblasts; FASP: filter aided sample preparation.

Despite many studies dealing with the isolation and mass spectrometric investigation of lysosomes, the protocols for their proteomic analysis have not been optimized to date, and sample preparation varies strongly between different datasets. In the current study, we used lysosomes isolated from mouse embryonic fibroblasts (MEFs) to systematically evaluate major steps of sample preparation and mass spectrometric analysis for lysosome-enriched fractions. We analyzed LC gradient lengths, solid phase extraction resins, peptide fractionation, and concentration approaches for lysosomes and lysosomal proteins in combination with protocols for proteolytic digestion (Fig. 1a, Table 1). Using these datasets, we generated a high confidence draft of the proteome of MEF lysosome enriched fractions, and a spectral library for their analysis by data independent acquisition (DIA).

**Table 1:** Samples analyzed by mass spectrometry. For all analyses, lysosomes isolated from MEFs were used. Different methods were applied for sample digestion, desalting, fractionation, liquid chromatography, and mass spectrometric data acquisition. Which method parameter  was compared with the individual *.raw files is indicated in the column “Method”. MEFs: mouse embryonic fibroblasts; CN: concentration by centrifugation; PN: concentration by precipitation; DDA: data dependent acquisition; DIA: data independent acquisition.

Sample	Source	Enriched Organelle	Method	Acquisition Strategy	Replicates
1 h	MEFs	Lysosome	Gradient	DDA	3
2 h	MEFs	Lysosome	Gradient	DDA	3
3 h	MEFs	Lysosome	Gradient	DDA	3
4 h	MEFs	Lysosome	Gradient	DDA	3
6 fractions	MEFs	Lysosome	Fractionation	DDA	3
3 fractions	MEFs	Lysosome	Fractionation	DDA	3
Unfractionated	MEFs	Lysosome	Fractionation	DDA	3
Stage Tip	MEFs	Lysosome	Desalting	DDA	3
Oasis	MEFs	Lysosome	Desalting	DDA	3
Sep-Pak	MEFs	Lysosome	Desalting	DDA	3
In-gel CN	MEFs	Lysosome	Digestion	DDA	3
In-gel PN	MEFs	Lysosome	Digestion	DDA	3
Urea CN	MEFs	Lysosome	Digestion	DDA	3
Urea PN	MEFs	Lysosome	Digestion	DDA	3
FASP CN	MEFs	Lysosome	Digestion	DDA	3
FASP PN	MEFs	Lysosome	Digestion	DDA	3
RapiGest CN	MEFs	Lysosome	Digestion	DDA	3
RapiGest PN	MEFs	Lysosome	Digestion	DDA	3
0.5 h	MEFs	Lysosome	Gradient	DIA	3
1 h	MEFs	Lysosome	Gradient	DIA	3
2 h	MEFs	Lysosome	Gradient	DIA	3
3 h	MEFs	Lysosome	Gradient	DIA	3
4 h	MEFs	Lysosome	Gradient	DIA	3

## Methods

### Cell culture and isolation of lysosomes

Mouse embryonic fibroblasts (MEFs) were cultured in Dulbecco’s Modified Eagle Medium (DMEM) supplemented with 10% fetal calf serum (FCS), 100 IU/mL penicillin, 100 µg/mL streptomycin, and 2 mM L-glutamine at 37 °C and 5% CO_2_. For lysosome isolation, 3 × 10^6^ cells were seeded per 10 cm plate and cultured in DMEM with 2.5% FCS for 72 h^7^. To each plate, 1 mL magnetite solution (EndoMAG40, Liquids Research, North Wales, UK) was added followed by 24 h incubation. Subsequently, the medium was exchanged, the cells were washed twice with 1x phosphate buffered saline (PBS), and a 24h  chase was performed in DMEM with 10% FCS. For harvesting, the cells were washed twice with ice-cold 1x PBS and scraped off the plate in 2 mL isolation buffer (250 mM sucrose, 10 mM HEPES/NaOH pH 7.4, 1 mM CaCl_2_,  15 mM KCl, 1 mM MgCl_2_, 1.5 mM MgAc, 1 mM dithiothreitol (DTT), 1x cOmplete EDTA-free protease inhibitor cocktail (Roche Diagnostics, Mannheim, Germany)) per plate. The cell suspension was homogenized with a 15 mL douncer, nuclei and intact cells were pelleted by centrifugation at 4 °C, 600 g for 10 min, and the post-nuclear supernatant was transferred to a new tube. This process was repeated and the post-nuclear supernatants were pooled. The resulting samples were then passed by gravity through a LS column located in a MidiMACS Separator (both Miltenyi Biotech, Auburn, CA). Subsequently, the column was washed with 5 mL isolation buffer, removed from the stand, and the lysosome fraction was eluted by a plunger in 2 × 1 mL isolation buffer. The efficiency of lysosome enrichment and lysosomal integrity was assessed using the β-hexosaminidase assay^7^: lysosome samples were incubated with the substrate 4-Nitrophenyl N-acetyl-β-D-glucosaminide in 0.1 M sodium citrate buffer (pH 4.6) containing 0.2% BSA with/without addition of 0.8% Triton X-100 (final concentration) at 37 °C for 15 min. The reaction was stopped by addition of 0.4 M glycine (pH 10.4) and the absorbance was measured at 405 nm. The protein concentration was determined using the DC protein assay (BioRad, Hercules, CA)^31^. For pelleting of intact lysosomes, the elution fraction was centrifuged at 4 °C, 20,000 g for 30 min, and the supernatant discarded. For protein precipitation, lysosomes were lyzed by addition of 1% Triton X-100 (final concentration), ice-cold chloroform/methanol (2:1, v/v) was added at a ratio of 5 to 1 (v/v), and the mixture was incubated on ice for 1 h. Following centrifugation at 4 °C, 20,000 g for 30 min, the liquid phases were discarded, the protein layer was washed with 1 mL ice cold methanol, and centrifuged at 4 °C, 14,000 g for 20 min and the supernatant discarded.

### In-gel digestion

Samples were denatured and reduced by addition of 1x modified Laemmli buffer^32^ (62.5 mM Tris-HCl, pH 6.8, 2% SDS, 10% glycerol, 5 mM DTT, 0.001% bromophenol blue) and incubation at 40 °C for 10 min. Proteins were alkylated with 20 mM acrylamide at room temperature (RT) for 30 min in the dark, loaded onto a 10% SDS gel, and electrophoresis was performed until the sample had migrated for ~1 cm into the separation gel. The gel was stained with Coomassie brilliant blue and the whole section of the gel containing the sample cut into ~1 mm^3^ cubes. In-gel digestion was performed as described elsewhere^33^. Briefly, the gel pieces were destained by 30% ACN/0.07 M NH_4_HCO_3_, dehydrated by 100% ACN, dried in a vacuum centrifuge, and digested with 1 µg trypsin (Promega, Madison, WI) in 0.1 M NH_4_HCO_3_ at 37 °C overnight. For the recovery of peptides, the supernatant was transferred to a new tube and the gel pieces were incubated consecutively with 0.1% TFA/50% ACN, 0.1 M NH_4_HCO_3_, and 100% ACN. The supernatants of the individual steps were pooled and dried using a vacuum centrifuge.

### Urea in-solution digestion

Samples were resuspended in 8 M urea/0.1 M TEAB^34^, and incubated at RT, 800 rpm for 45 min. Proteins were reduced with 5 mM DTT (final concentration) at 56 °C, 800 rpm for 25 min, alkylated with 20 mM acrylamide at RT for 30 min in the dark, and the reaction was quenched by addition of 5 mM DTT. Subsequently, the concentration of urea was reduced to 4 M, rLys-C (Promega) added at an enzyme to protein ratio of 1 to 100, and the sample incubated at 37 °C overnight. Subsequently, the samples were diluted to a final concentration of 1.6 M urea with 0.1 M TEAB, trypsin was added at an enzyme to protein ratio of 1 to 100, and the sample was incubated at 37 °C for 10 h. Finally, the samples were acidified using acetic acid (AcOH, 0.1% final concentration).

### Filter aided sample preparation (FASP)

Samples were solubilized in 20 µL 4% SDS/0.1 M Tris-HCl, pH 7.6 at 40 °C for 5 min, and reduced with 0.1 M DTT (final concentration) at 56 °C for 5 min. Subsequently, FASP digestion was performed as described elsewhere^35^ with slight modifications. Briefly, samples were mixed with 200 µL of UA (8 M urea/0.1 M TEAB) and added to a filter unit (Microcons, 30 kDa cut off, Merck Millipore, Darmstadt, Germany). Subsequently, buffers were exchanged with UA solution by two consecutive centrifugation steps at RT, 14,000 g for 15 min, and proteins were alkylated by addition of 100 µL AA solution (0.05 M acrylamide in UA)^36^ at RT for 20 min. The filter units were then washed twice with 100 µl of 0.05 M NH_4_HCO_3_ by centrifugation at 14,000 g for 10 min. Subsequently, 60 µl of 0.05 M NH_4_HCO_3_ and 10 µl of trypsin solution (0.1 µg/µl) were added and the sample was incubated in a wet chamber at 37 °C overnight. The digested peptides were recovered from the filter units by centrifugation at 14,000 g for 10 min and subsequent elution with 50 µl 0.5 M NaCl followed by centrifugation. Eluted peptides were acidified with AcOH (0.1% final concentration).

### RapiGest in-solution digestion

Samples were solubilized in 1% RapiGest (Waters, Milford, MA)/0.1 M NH_4_HCO_3_, pH 7.8 at 37 °C for 45 min and diluted 1:1 with 0.1 M NH_4_HCO_3_. Proteins were reduced with DTT (5 mM final concentration) at 56 °C for 25 min, alkylated with acrylamide (20 mM final concentration) at RT for 30 min, and the reaction was quenched by addition of 5 mM DTT. Samples were further diluted to a final concentration of 0.1% RapiGest with 0.1 M NH_4_HCO_3_ (protein concentration: 1 µg/µL). Proteins were digested with trypsin (enzyme to protein ratio of 1 to 100) at 37 °C overnight. The next day, RapiGest was hydrolyzed by the addition of 1% TFA (final concentration) and incubation at 37 °C, 800 rpm for 30 min, followed by its precipitation at RT, 20,000 g for 10 min. The supernatants were transferred to new tubes.

### Desalting of peptides

Peptides were desalted either using 10 mg Oasis HLB cartridges (Waters), 10 mg Sep-Pak C_18_ 1 cc Vac cartridges (Waters), or Stage Tips^37^ assembled with 3 M™ Empore™ C_18_ reversed phase material (3 M, St. Paul, MN). Oasis and Sep-Pak cartridges were equilibrated with MeOH, 70% ACN/0.5% AcOH, and 0.5% AcOH, the samples loaded, and the cartridges washed three times with 1 mL 0.5% AcOH. Peptides were eluted consecutively with 500 µL 30% ACN/0.5% AcOH, 300 µL 50% ACN/0.5% AcOH and 300 µL 70% ACN/0.5% AcOH and the eluate fractions were combined. In case of C_18_ Stage Tips, tip columns were prepared with 3 layers of C_18_ disks in a 200 µL pipette tip. Tip columns were equilibrated sequentially with MeOH, 80% ACN/0.5% AcOH, and 0.5% AcOH by centrifugation at 600 g for 1 min. Acidified samples were loaded onto the column, washed with 100 µL 0.5% AcOH, and peptides were eluted twice with 20 µL of 80% ACN/0.5% AcOH by centrifugation at 600 g for 1min. Eluate fractions were dried using a vacuum centrifuge.

### Pipette tip based strong anion exchange (SAX) fractionation of peptides

SAX fractionation was performed as described elsewhere^38^. Briefly, a pipet tip SAX column was assembled using 12 disks of Empore Anion-SR material (3 M) and C_18_ Stage Tips were generated using 3 disks of Empore C_18_ material. SAX buffers were composed of 20 mM AcOH, 20 mM phosphoric acid, and 20 mM boric acid. The pH of the individual solutions was adjusted to pH 11, 8, 6, 5, 4, and 3 by addition of NaOH. Subsequently, NaCl was added to the final elution buffer (pH 3) at a concentration of 0.25 M. SAX columns were equilibrated by sequential addition of 100 µL of MeOH, 1 M NaOH, and loading buffer (pH 11), each in combination with subsequent centrifugation at 7,000 g for 3 min. Stage Tips were equilibrated with 100 µL of MeOH, 80% ACN/0.5% AcOH, and water. The dried peptide samples were resuspended in 200 µL pH 11 buffer, loaded on the SAX column, and fractionation was performed by centrifugation at 7,000 g for 3 min for each step. The flow-through and the individual elution fractions were captured on the C_18_ Stage Tips, which were further washed with 100 µL of 0.5% AcOH, and eluted by 80% ACN/0.5% AcOH. The desalted peptides were dried using a vacuum centrifuge.

### UHPLC-MS/MS data acquisition

Analyses were performed using a Dionex Ultimate 3000 system coupled to an Orbitrap Fusion Lumos mass spectrometer (both Thermo Scientific, Bremen, Germany). Columns were produced in-house as follows: 50 cm spray tips were generated from 360 μm outer diameter/100 μm inner diameter fused silica capillaries using a P-2000 laser puller (Sutter Instruments, Novato, CA) and packed with 1.9 μm Reprosil AQ C_18_ particles (Dr. Maisch, Ammerbuch-Entringen, Germany). Peptides were resuspended in 5% ACN/5% FA and loaded on the analytical column at a flow rate of 600 nL/min, 100% solvent A (0.1% FA in water). Subsequently, the separation was performed at a flow rate of 300 nL/min with 60, 120, 180, and 240 min linear gradients from 5–35% solvent B (95% ACN/0.1% FA). Survey spectra were acquired in the Orbitrap mass analyzer with a mass range of m/z 375–1,575 at a resolution of 60,000. MS/MS fragmentation was performed in the data dependent acquisition mode for charge states between 2–4 by HCD and data were acquired in the Orbitrap at a resolution of 30,000. The cycle time was set to 5 s and the precursor isolation width to 1.6 m/z using the quadrupole. For MS1 and MS2 scans, the automatic gain control (AGC) was set to 4 × 10^5^ and 5 × 10^5^, respectively. Fragmented ions were excluded from further fragmentation for 30 s, 60 s, 90 s, and 120 s, respectively, for the four different gradient lengths. For data-independent acquisition (DIA) analysis of the samples, the following method was applied: One MS1 scan with a resolution of 120,000, an AGC target setting of 5 × 10^5^, and a maximum injection time of 20 ms covering a mass range of 350 to 1,200 m/z was performed followed by 18 to 58 static DIA scans depending on the gradient length (0.5 h: 18 scans, 1 h: 24 scans, 2 h: 36 scans, 3 h: 47 scans, 4 h: 58 scans). The isolation window widths were adjusted for each gradient length to cover the same mass range as the MS1 scan including a 0.5 m/z overlap (0.5 h: 47.7 m/z, 1 h: 35.9 m/z, 2 h: 24.1 m/z, 3 h: 18.6 m/z, 4 h: 15.2 m/z). The DIA scans were acquired with a resolution of 30,000, an AGC target setting of 1 × 10^6^, and a maximum injection time of 60 ms. The HCD collision energy was set to 27% and the resulting cycle times based on the window designs were as follows for the individual methods: 0.5 h: 1.89 s, 1 h: 2.34 s, 2 h: 3.44 s, 3 h: 4.46 s and 4 h: 5.45 s.

### Data analysis – data dependent acquisition (DDA)

Thermo *.raw data were analyzed with Proteome Discoverer 2.2 (Thermo Fisher Scientific, Bremen, Germany) in combination with Mascot (www.matrixscience.com). For database searching, Uniprot *Mus musculus* (release 2019_04, 54,425 entries) in combination with the cRAP database (ftp://ftp.thegpm.org/fasta/cRAP/crap.fasta) including common contaminants was used with the following parameters: variable modifications: oxidation of methionine, acetylation of protein N-termini; fixed modification: propionamide at cysteine; mass tolerance: 10 ppm for precursor ions, 50 mmu for fragment ions; enzyme: trypsin except proline was the next amino acid; missed cleavage sites: 2. Data were filtered with a false discovery rate (FDR) of 1% at the peptide level using Percolator and proteins were exported with a FDR of 1%. Label free quantification was performed using the Minora feature detector node in Proteome Discoverer.

### Data processing – data dependent acquisition (DDA)

Only high confidence identifications were exported to MS Excel for further analyses. Numbers of lysosomal proteins were determined from protein files by comparison to a list of confirmed lysosomal proteins (figshare deposit^39^: Table 9_Lysosomal Protein List) generated by merging of a manually curated bona fide list^6–8,10,19,40–42^ and a publicly available gene ontology database (www.pantherdb.org). Peptide spectral match (PSM) and peptide numbers were determined from the PSM files. For label free quantification, proteins with an average intensity ratio of log2 > 1 or log2 < 0.5 and a p-value < 0.05 were considered to be significantly over-/underrepresented. Missed cleavage rates for the individual digestion methods were determined from the PSM files by calculating the number of peptides with one or more missed cleavage sites and normalization on the total number of identified peptides. For identification of semi-tryptic peptides, database searches were repeated with enzyme specificity set to semi-trypsin, followed by normalization of identified semi-tryptic peptides on the number of total peptides identified.

### Data analysis – data independent acquisition (DIA)

DIA data were analyzed using the Pulsar^43^ algorithm available in Spectronaut (Version: 13.2.19, Biognosys, Schlieren, Switzerland). A spectral library was generated based on the same parameters as defined for the analysis of the DDA data with Proteome Discoverer 2.2 except the mass tolerances, which were assigned dynamically by the Pulsar algorithm. To build the library, 3 to 6 fragment ions per peptide were selected based on their intensity. All DIA data were analyzed using this library in combination with the default settings of Spectronaut. For retention time alignment, the high precision iRT concept was applied^43^. Peak extraction windows, as well as the mass tolerances for the matching of precursor and fragment ions, were determined automatically by Spectronaut. For peak detection, a minimum requirement of 3 fragment ions was defined, whereby precursor information was only used to enhance peak detection. Data normalization was performed using local regression localization with enabled interference correction. Data were filtered at 1% FDR on the peptide precursor and protein level applying a Q-value cut-off of <0.01^44^. The generated Spectronaut project file can be viewed using the freely available Spectronaut viewer.

## Data Records

The mass spectrometry data and analysis files have been deposited to the ProteomeXchange Consortium (http://www.proteomexchange.org) via the PRIDE partner repository^38^. The DDA dataset includes 75 *.raw files representing all experimental conditions (Gradient tests: 4 conditions; Desalting tests: 3 conditions; Fractionation tests: 3 conditions; Digestion tests: 8 conditions) from three experimental replicates each. The fractionation dataset includes *.raw files for each individual fraction. The DIA dataset includes 15 *.raw files comprising 0.5, 1, 2, 3 and, 4 h gradient length tests with three replicates each. Furthermore, the dataset includes the result files originating from Proteome Discoverer (7x .pdResult files, 7x pepXML search result files, 7x .pdStudy files and 15x MSF files) and one result file from Spectronaut. In addition, the protein list data from the .pdResult files are available as excel tables for each experiment. These individual analyses, as well as the list of confirmed lysosomal proteins, can be accessed through a figshare deposit^39^.

## Technical Validation

In order to provide a reproducible starting material for all analyses, we generated a large batch of lysosome enriched fractions from forty-eight 10 cm dishes of mouse embryonic fibroblasts (MEFs) employing superparamagnetic iron oxide nanoparticles (SPIONs)^7,15^. To assess the purity and the amount of intact lysosomes, we performed enzyme activity assays for β-hexosaminidase, a hydrolase residing in the lysosomal lumen. We were able to recover ~80% of the intact lysosomes contained in the starting material and the enrichment efficiency of the magnetic column was 62% (Fig. 1b). In the eluate fraction, 77% of lysosomes were intact (determined by the difference in enzyme activity with/without Triton X-100, Fig. 1b). When enriched by SPIONs, lysosomes are eluted from a magnetic column in a rather big volume and therefore the sample needs to be concentrated. For this purpose, and the removal of the isolation buffer which may interfere with tryptic digestion, we employed two strategies: 1) the precipitation of all proteins by chloroform/methanol (precipitation samples, PN); and 2) the concentration of intact lysosomes by centrifugation (centrifugation samples, CN). For each approach, we prepared 24 identical aliquots, which were stored at −80 °C until further use and determined the protein concentration for one representative aliquot.

### Impact of LC gradient length on protein identification

**Fig. 2:**
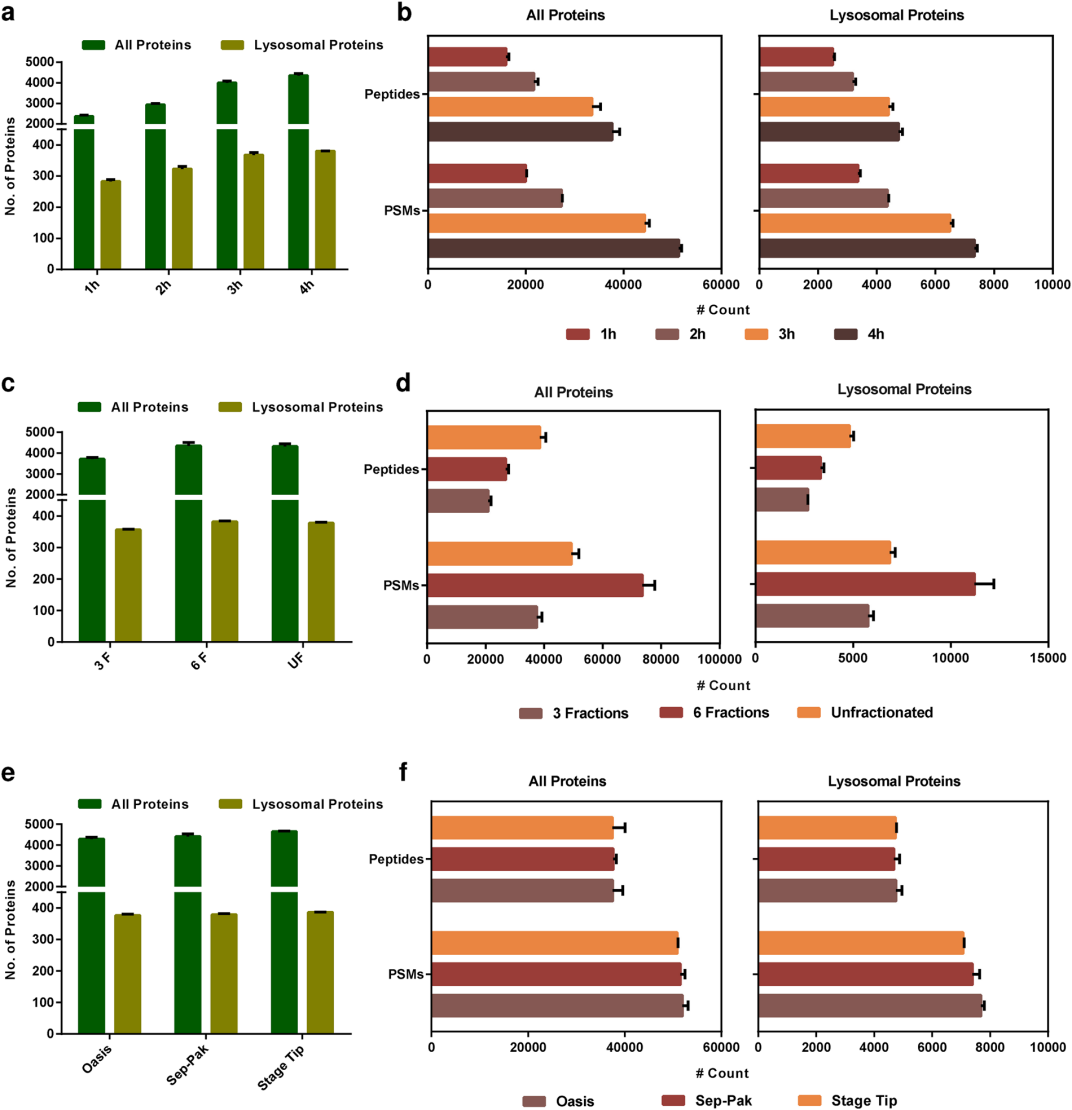
Evaluation of the impact of gradient length, sample fraction, and desalting on protein identification. **(a)** Analysis of unfractionated tryptic digests of lysosome enriched fractions by LC-MS/MS using different gradient lengths (1 h, 2 h, 3 h, and 4 h). Shown are the average numbers of identified total and lysosomal protein groups. **(b)** Average numbers of PSMs and total peptides identified with different gradient lengths for total and lysosomal protein groups. **(c)** Analysis of the impact of peptide fractionation on the number of identified total and lysosomal protein groups. **(d)** Average numbers of PSMs and total peptides identified for the different fractionation methods. **(e)** Evaluation of the impact of different desalting approaches on the numbers of identified proteins groups using Stage Tip- or cartridge-based formats. **(f)** Average numbers of PSMs and total peptides for the different desalting methods. Shown are average values of 3 independent replicates + standard deviation. 3 F: 3 fractions; 6 F: 6 fractions; UF: unfractionated; PSMs: peptide spectral matches.

Initially, we determined the impact of the LC gradient on the identification rates of peptides and proteins by analyzing 1 µg of urea digested PN sample in triplicates with four different gradient lengths (1 h, 2 h, 3 h, and 4 h). In comparison to 1 h gradients (2367 protein groups on average, 1962 identified in all 3 replicates), an increase in analysis time resulted in an average gain of 24%, 69%, and 84% proteins groups for the 2 h, 3 h, and 4 h gradients, respectively (Fig. 2a, and in figshare deposit^39^: Table 1_Proteins_Gradient Length). We further focused on a subset of lysosomal and lysosome-associated proteins (figshare deposit^39^: Table 9_Lysosomal Protein List) in the dataset. The effects observed for this group of proteins were less pronounced compared to the whole protein population with a maximal increase of 34% (Fig. 2a). When we also took the reproducibility of identification into account, however, the difference between the 1 h and 4 h gradient increased to 47% (only proteins identified in all 3 replicates). Furthermore, the number of peptide spectral matches and unique peptides assigned to lysosomal proteins raised by 2.2 fold and 1.9 fold, respectively (Fig. 2b). Also for the whole dataset, we observed similar trends but with slightly higher fold-change values (Fig. 2a,b). Compared to the total population of proteins identified in the dataset, the reproducibility of identification for lysosomal proteins was higher for all gradient lengths tested, reaching to a reproducibility of 94% of proteins detected in all three replicates for the analysis with 4 h gradients.

### Impact of peptide fractionation and desalting on protein and peptide identification

With ~4,350 protein groups on average for the 4 h measurements, the comparison of LC gradients revealed a higher complexity of the lysosome-enriched samples than anticipated. We therefore evaluated, if further fractionation could improve identification rates as it allows for higher amounts of starting material and the individual fractions are of lower complexity. We employed SAX-tip based fractionation^35^ generating 3 or 6 fractions in three independent replicates and analyzed them with linear gradients of 60 min, as well as an unfractionated sample with a 4 h gradient (figshare deposit^39^: Table 2_Proteins_Fractionation Methods). With regard to the total number of identified protein groups, the sample divided into 6 fractions resulted in virtually similar numbers compared to the unfractionated sample (4,349 and 4,323 protein groups on average, respectively) while the sample divided into 3 fractions yielded on average only 3,719 protein groups (Fig. 2c). When considering just the proteins identified in all three replicates, the unfractionated sample outperformed both fractionation methods. For our subset of bona fide lysosomal proteins, we observed a similar trend with the unfractionated sample yielding the highest number of identified lysosomal proteins and the most reproducible results (Fig. 2c). These results indicate that the 4 h gradient is sufficient for the complexity of the lysosomal fractions and no under-sampling occurs. With respect to the number of identified peptide spectral matches (PSMs) for total and lysosomal proteins, the sample divided into 6 fractions yielded the best results, and for numbers of unique peptides identified, the unfractionated sample performed best (Fig. 2d).

In order to evaluate if desalting influences the identification of peptides and proteins, we performed solid phase extraction with three different resins and compared the results. This included a tip-based format using C_18_ Stage Tips^37^ as well as two solid phase extraction cartridges containing different stationary phases: Oasis HLB (Hydrophilic-Lipophilic Balance) cartridges, and Sep-Pak C_18_ cartridges. Using the urea digested PN sample, we desalted 10 µg of peptides with Stage Tips, and with both cartridge types 40 µg of peptides in triplicates followed by analysis of 1 µg each with 4 h gradients (figshare deposit^39^: Table 3_Proteins_Desalting Methods). Desalting with Stage Tips resulted in the highest number of lysosomal and total protein groups identified in all 3 replicates, followed by Sep-Pak and Oasis, which both delivered similar results (Fig. 2e). For both the whole dataset and the lysosomal proteins, however, the Oasis cartridges slightly outperformed both other approaches concerning the average number of PSMs and unique peptides (Fig. 2f). Concerning reproducibility, Stage Tip-based desalting outperformed both other methods.

### Investigation of sample concentration and digestion procedures

Eluate fractions obtained from SPIONs enrichment are often highly diluted and it is necessary to further concentrate the contained lysosomes (or lysosomal proteins). Dependent on the subsequent experiments, it may be required to preserve the organelles’ integrity (for example for enzymatic assays) excluding the application of denaturing protein precipitation (PN) approaches. Furthermore, precipitation could result in protein aggregates which may not be fully re-solubilized during sample preparation for proteomic analysis. Therefore, the pelleting of intact lysosomes by centrifugation (CN) is an attractive alternative. This approach should not result in any solubilization issues and may, as a positive side effect, lead to the depletion of unspecifically enriched soluble proteins, which will not be pelleted. However, CN may not succeed in the recovery of all lysosomes (e.g. such damaged during isolation) and proteins interacting weakly with the lysosomal surface may be lost. Furthermore, due to the lack of a denaturation step, lysosomal proteases may retain residual activity during proteolytic digestion possibly influencing the results obtained from these samples.

To compare these two individual concentration approaches, we pelleted intact lysosomes by centrifugation (CN) or chemically precipitated proteins (PN) contained in the lysosome enriched fractions. We combined both approaches with four commonly used methods for proteolytic digestion including in-gel digestion, filter aided sample preparation (FASP), and in-solution digestion using either RapiGest or urea, resulting in 8 different combinations in total (Fig. 1a). For each combination of sample concentration and digestion, we prepared three independent replicates. 10 µg of peptides were desalted by Stage Tips, and 1 µg each was analyzed with a 4 h gradient (figshare deposit^39^: Table 4_Proteins_Digestion Methods).

**Fig. 3:**
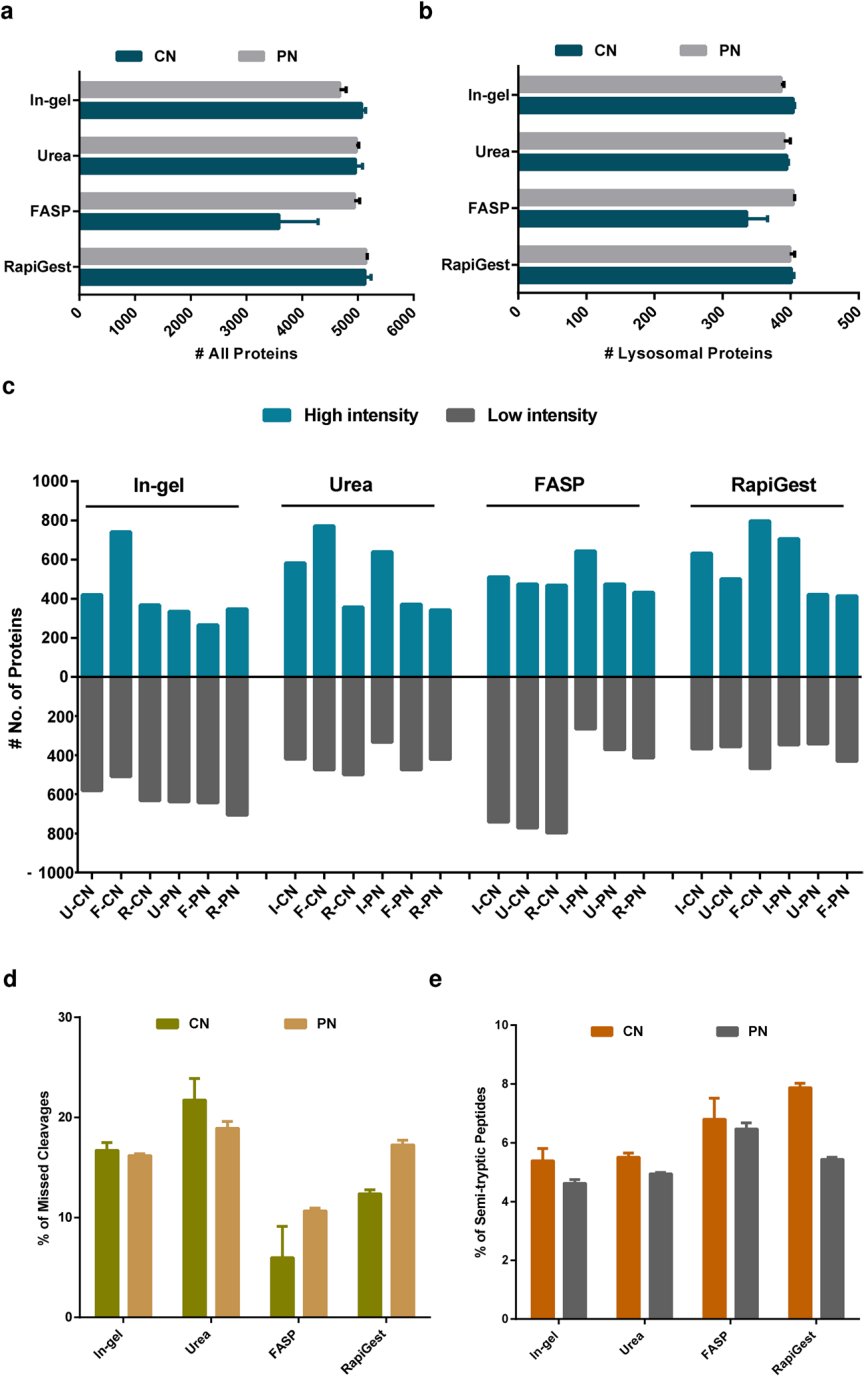
Evaluation of concentration and digestion methods. **(a)** Average numbers of identified protein groups for the individual combinations of concentration and digestion methods. **(b)** Average numbers of identified lysosomal protein groups for the individual combinations of concentration and digestion methods. **(c)** Number of proteins significantly over-/underrepresented for binary comparisons of the individual datasets (fold-change up or down regulation of ≥2 and p-value < 0.05). **(d)** Average numbers of missed cleavage sites for the individual combinations of concentration and digestion methods. **(e)** Average percentage of semi-tryptic peptides for the individual combinations of concentration and digestion methods. Shown are average values of 3 independent replicates + standard deviation (**a**,**b**,**d**,**e**). PN: Precipitation; CN: centrifugation; U-CN: Urea CN; F-CN: FASP CN; R-CN: RapiGest CN; U-PN: Urea PN; F-PN: FASP PN; R-PN: RapiGest PN; I-CN: In-gel CN; I-PN: In-gel PN.

For the in-solution digestion with urea and RapiGest, we observed virtually no differences between both the digestion and the concentration strategies concerning the number of identified lysosomal and total protein groups (Fig. 3a,b). For sample preparation by FASP, the CN sample resulted in a markedly reduced number of total as well as lysosomal proteins, with high variability in total protein numbers between the individual replicates. In-gel digested samples yielded slightly better results for the CN samples for both lysosomal and total proteins. To further investigate differences for the individual approaches, we performed label free quantification using a combined database search (figshare deposit^39^: Table 5_LFQ_Digestion Methods). We filtered for protein groups identified with all eight workflows and performed binary comparisons for the individual digestion approaches within the same sample concentration setup (PN or CN). We then determined for each combination the number of proteins which were overrepresented in a given sample with a p-value < 0.05 and fold-change of ≥2 (Fig. 3c, and in figshare deposit^39^: Table 5_LFQ_Digestion Methods). For each individual approach, a specific subset of proteins was overrepresented suggesting that the choice of sample preparation should be adapted if specific proteins are of special interest. Furthermore, these data suggest that results from published studies employing different digestion strategies can be compared in a qualitative but not a quantitative way. To further investigate the regulated protein populations, we performed GO analyses for proteins which were significantly up- or downregulated, the results can be found in our figshare deposit^39^: Table 5_LFQ_Digestion Methods.

As protein precipitation may result in aggregates which could influence the efficiency of proteolytic digestion, we further investigated the percentage of missed cleavage sites (Fig. 3d). While in-gel digestion resulted in similar rates for both the PN and the CN sample, we observed for FASP and RapiGest higher numbers of missed cleavages for PN. For the urea digested sample, the CN sample resulted in a slightly higher percentage of missed cleavage sites than PN and in general in a less efficient cleavage than for the other approaches.

Lysosomes contain >20 different proteases of which ~50% belong to the family of the cathepsins, catalyzing the degradation of a wide variety of proteins^45^. Dependent on their concentration, both urea and RapiGest retain the activity of the proteases Lys-C and trypsin which are used for mass spectrometry sample processing. It was shown before, that also in pH values higher than those usually present in the lysosomal lumen, cathepsins can be enzymatically active^46^. We, therefore, investigated if the CN samples still contained active lysosomal proteases, since no protein precipitation step was part of this protocol. As active cathepsins should result in peptides cleaved at other sites than arginine or lysine (for digests with trypsin and Lys-C), we performed database searches for semi-tryptic peptides (figshare deposit^39^: Table 6_Semi-tryptic peptides_Digestion Methods). For all digestion methods, we observed a slight increase in semi-tryptic peptides for those concentrated by CN (Fig. 3e). This was especially pronounced for samples digested in RapiGest for which ~1,600 additional semi-tryptic peptides were identified in the CN relative to the PN sample (increase of 67%). The markedly higher difference for digests carried out in RapiGest suggests that certain lysosomal proteases may still be enzymatically active in these samples.

**Fig. 4:**
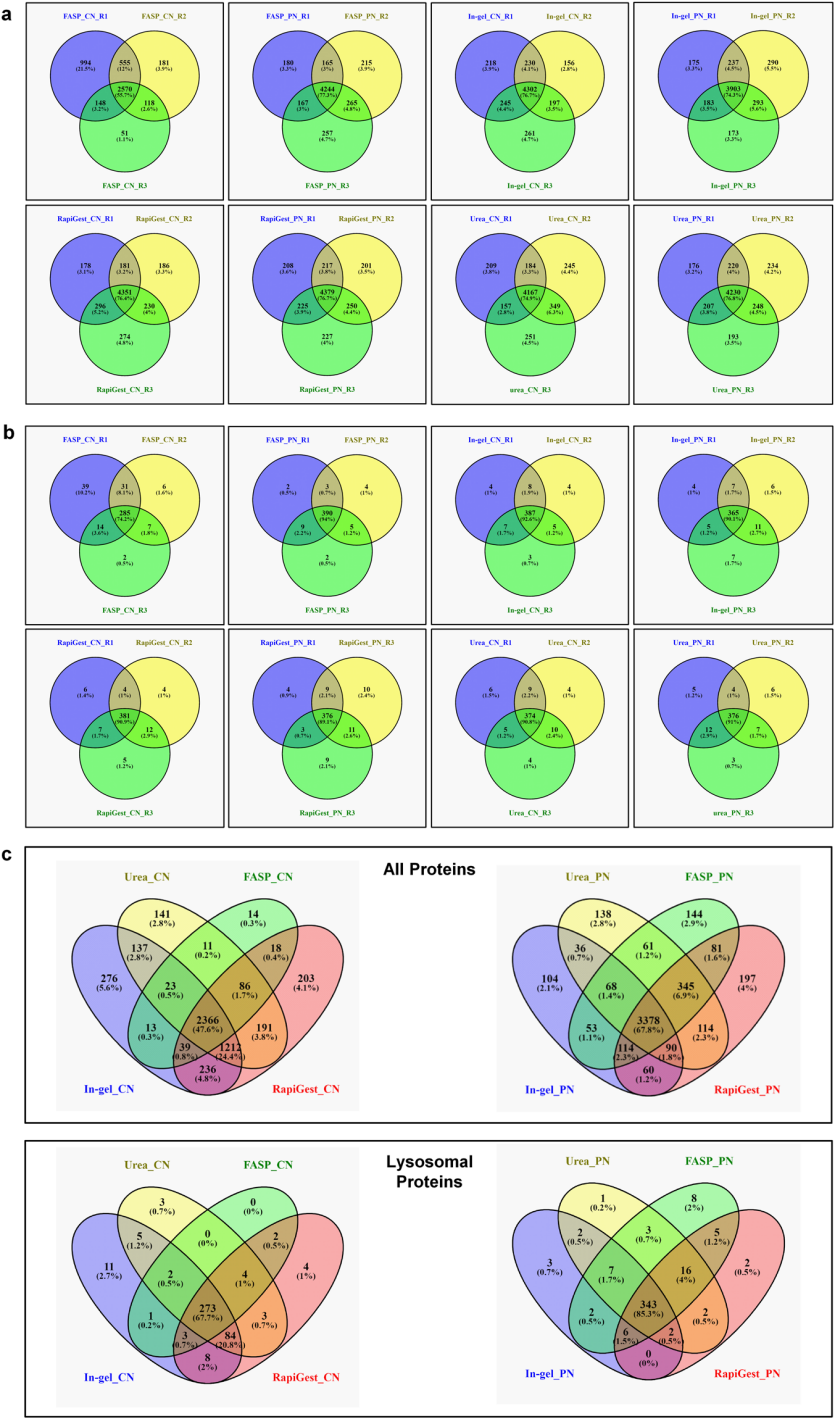
Reproducibility of protein identification for the individual sample preparation methods. **(a)** Common and unique total proteins among individual replicates for each combination of sample concentration approach and digestion method. **(b)** Common and unique lysosomal proteins among individual replicates for each combination of sample concentration approach and digestion method. **(c)** Overlap of identified total or lysosomal proteins for the combination of a specific type of sample concentration and digestion method. PN: concentration by precipitation; CN: concentration by centrifugation.

Concerning reproducibility among individual replicates, all approaches (with the exception of the FASP CN samples) performed similar resulting in an approximate overlap of 75% for total proteins and ~90% for lysosomal proteins (Fig. 4a,b). For the individual digestion strategies within each concentration method, the PN samples showed with 68%/85% a better performance than the CN samples (48%/68%) for the whole population of proteins and such located at the lysosome, respectively (Fig. 4c).

### Draft map of the mouse embryonic fibroblast (MEF) lysosomal proteome

**Fig. 5:**
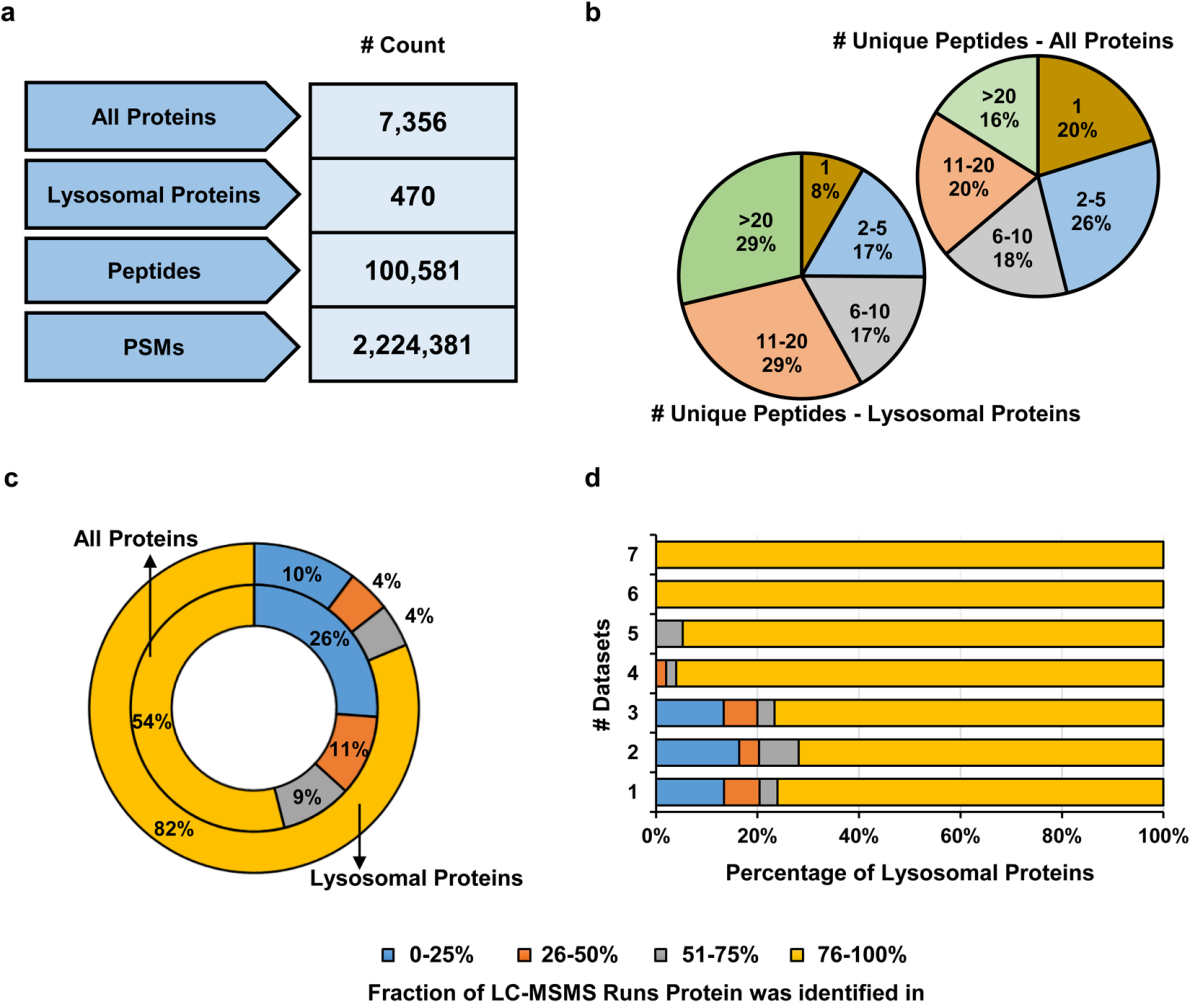
Draft map of the MEF lysosomal proteome. **(a)** Number of identified proteins, lysosomal proteins, peptides, and PSMs from the combined database search of 39 × 4 h gradient measurements. **(b)** Distribution of total and lysosomal proteins identified with a specific number of unique peptides. **(c)** Reproducibility of proteins and lysosomal proteins identified across the individual analyses. Shown is the percentage of proteins identified within a certain fraction of the 39 LC-MS/MS runs analyzed. **(d)** Correlation of the identification rate of lysosomal proteins and their occurrence in published datasets.

Utilizing measurements from all conditions, for which we analyzed the samples with 4 h gradients (39 LC-MS/MS runs in total), we performed a combined database search in order to assemble a draft map of the MEF lysosomal proteome (figshare deposit^39^: Table 7_Combined_Database Search_4h Gradient Length). In total, we identified 7,356 proteins from 100,581 peptides and 2,224,381 high confidence PSMs (Fig. 5a). For the unique peptides identified (83,619 in total), we observed a trend towards higher numbers for known lysosomal proteins (75% identified with >5 unique peptides) compared to the whole dataset (54% identified with >5 unique peptides (Fig. 5b). On the protein level, we were able to identify 470 out of 740 proteins of known lysosomal origin in total. Of these proteins, 82% were detected in >75% of LC-MS/MS runs while for the whole dataset only 54% were identified at the same rate (Fig. 5c). We further matched the detection rate of lysosomal proteins with their occurrence in published datasets (Fig. 5d, figshare deposit^39^: Table 9_Lysosomal Protein List). We observed a correlation of the number of datasets which list a given proteins as lysosomal and the identification rate in our data: the likelihood to be reproducibly detected in our analyses increases with the number of published datasets including the protein. Taken together, this dataset presents to our knowledge the so-far most extensive analysis of lysosomes from a single cell type identifying a highly reproducible core proteome for lysosome enriched fractions from MEFs.

### Analysis of the MEF lysosomal proteome by data independent acquisition (DIA)

**Fig. 6:**
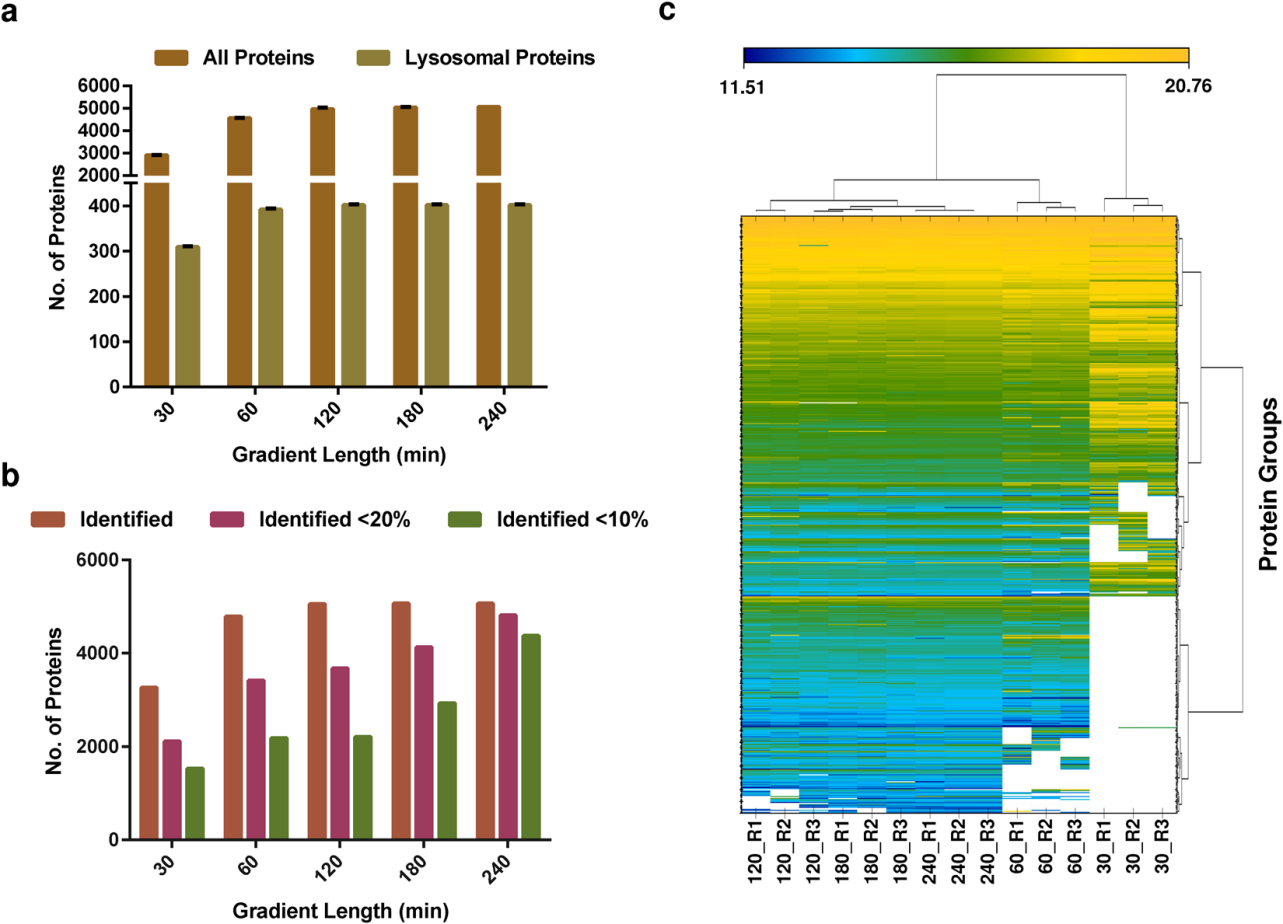
Analysis of the MEF lysosomal proteome by data independent acquisition. **(a)** Average numbers of total and lysosomal proteins identified for different gradient lengths. **(b)** Numbers of total and lysosomal proteins determined with values below a certain coefficient of variation (CV) among the individual replicates. **(c)** Unsupervised clustering of protein intensities determined for the different individual gradient lengths. Each column represents the normalized protein intensities for an individual replicate. In (**a**), average values of 3 independent replicates + standard deviation are shown. CV: coefficient of variation.

In order to facilitate an efficient quantification of lysosome enriched fractions from MEFs in future studies by DIA, we generated a spectral library based on our dataset obtained from the combined searches by Proteome Discoverer. We imported the PD result file into the PulsarX algorithm integrated into the Spectronaut software and generated a spectral library covering 7421 proteins, 98,371 peptides, and 118,269 precursors^38^. We then analyzed 1 µg of urea in-solution digested lysosome enriched samples in 3 replicates with five different gradient lengths by DIA (30, 60, 120, 180 and 240 min, (figshare deposit^39^: Table 8_Proteins_Gradient length_DIA). While, not surprisingly, the 240 min gradient resulted in the highest number of protein identifications and the 30 min gradient in the lowest (Fig. 6a), the differences were much less pronounced as for the DDA measurements (Fig. 2a). Furthermore, when comparing gradient lengths ≥120 min, we virtually did not observe any differences in numbers of identified proteins. When we assessed the reproducibility of signal intensities for label free quantification, however, we found a continuous increase of proteins with low coefficients of variation (<10% CV) with the 240 min gradients delivering superior results (Fig. 6b). Finally, we visualized differences in protein identification and abundance for the individual DIA analyses clustering the data in a row and column-wise manner for all data points with high confidence (Fig. 6c). We observed a highly reproducible clustering of intensities among the independent biological replicates and highly similar profiles among the gradients with ≥120 min.

## Usage Notes

For all analyses, the identified protein groups including the most important information can be found in the respective table in the figshare collection^39^. Furthermore, for the analysis of missed cleavage sites, the individual peptide sequences are provided in our figshare deposit^39^ Table 6_Semi-tryptic Peptides_Digestion methods. If more details, like the exact peptide sequences assigned to a given protein in a specific analysis, are desired, the Proteome Discoverer (PD) result files can be accessed through the public repository^38^. For accession of these files, the Proteome Discoverer Software can be obtained from www.thermofisher.com. Furthermore, the PD study files are included which can be used to re-analyze the *.raw files with different parameters. If an analysis by a different algorithm is desired, the provided *.raw files can be analyzed with any other mainstream proteomic data analysis software. For manual analysis of the *.raw files, tools like Xcalibur or FreeStyle from Thermo Fisher Scientific can be used or freeware such as MSFileReader or the ProteoWizard toolkit.

A unique value of the dataset presented in this study is related to the planning of experiments for the analysis of lysosomes from MEFs by mass spectrometry. If the analysis of a given lysosomal or lysosome-associated protein in these cells is desired, it is possible to determine from the individual datasets which lysosome concentration method, proteolytic digestion strategy, desalting approach, fractionation method, and gradient length should be used to obtain an optimal result. For the development of targeted mass spectrometry assays for mouse samples, high confidence peptides and their fragment ions can be extracted from our combined dataset. This allows assessing how reproducible the identification of these peptides is across different experimental conditions, if the signal intensity is sufficient, and which fragment ions can be utilized for SRM/MRM assay design. Based on the information in figshare deposit^39^: Table 5_LFQ_Digestion Methods, it can furthermore be assessed if a certain digestion method results in higher intensities for the protein(s) of interest. Finally, the spectral library which was generated for the DIA analyses can be readily used for the analysis of lysosome-enriched MEF samples with the algorithm Spectronaut (www.biognosys.com). If analysis with other algorithms is desired, the data can be re-exported from the available PD study in the desired format. The definition of a high confidence lysosomal proteome by combination of 39 individual LC-MS/MS analyses presents, to our knowledge, the largest analysis of isolated MEF lysosomes so-far. This resource is valuable for the identification of proteins which are of potential lysosomal origin in MEF cells covering such which have been proposed to be located at the lysosome and such which have not been assigned to the lysosome yet.

Taken together, this dataset presents a toolbox for the conceptualization of experiments for the analysis of lysosome enriched samples from MEFs, and a valuable resource for the targeted analysis of lysosomal proteins in mouse samples.

## Data Availability

DDA data were analyzed with Proteome Discoverer 2.2 and DIA data were analyzed with Spectronaut (Version: 13.2.19). Data comparison was performed with Microsoft Excel 2016, GraphPad Prism 6.07, and Venny (https://bioinfogp.cnb.csic.es › tools › venny).

## References

[1] Ballabio A (2016). The awesome lysosome. EMBO Mol. Med..

[2] Ballabio A, Gieselmann V (2009). Lysosomal disorders: from storage to cellular damage. Biochim. Biophys. Acta Mol. Cell Res..

[3] Lim C-Y, Zoncu R (2016). The lysosome as a command-and-control center for cellular metabolism. J. Cell Biol..

[4] Fraldi A, Klein AD, Medina DL, Settembre C (2016). Brain disorders due to lysosomal dysfunction. Annu. Rev. Neurosci..

[5] Davidson SM, Vander Heiden MG (2017). Critical functions of the lysosome in cancer biology. Annu. Rev. Pharmacol. Toxicol..

[6] Geladaki A (2019). Combining LOPIT with differential ultracentrifugation for high-resolution spatial proteomics. Nat. Comm..

[7] Thelen, M., Winter, D., Braulke, T. & Gieselmann, V. in *Methods in Molecular Biology* Vol. 1594 (eds. Öllinger, K. & Appelqvist, H.) 1–18 (Humana Press, 2017). 10.1007/978-1-4939-6934-0_1 28456973

[8] Itzhak DN, Tyanova S, Cox J, Borner GHH (2016). Global, quantitative and dynamic mapping of protein subcellular localization. Elife.

[9] Wattiaux R, Wattiauxdeconinck S, Ronveauxdupal MF, Dubois F (1978). Isolation of rat-liver lysosomes by isopycnic centrifugation in a metrizamide gradient. J. Cell Biol..

[10] Chapel A (2013). An Extended Proteome Map of the Lysosomal Membrane Reveals Novel Potential Transporters. Mol. Cell. Proteom..

[11] Schroeder B (2007). Integral and associated lysosomal membrane proteins. Traffic.

[12] Zhang, H., Fan, X., Bagshaw, R., Mahuran, D. J. & Callahan, J. W. in *Methods in Molecular Biology* Vol. 432 (eds. Pflieger, D. & Rossier, J.) 229–241 (Humana Press, 2008). 10.1007/978-1-59745-028-7_16 18370022

[13] Leighton F (1968). Large-scale separation of peroxisomes mitochondria and lysosomes from livers of rats injected with Triton WR-1339- improved isolation procedures automated analysis biochemical and morphological properties of fractions. J. Cell Biol..

[14] Diettrich O, Mills K, Johnson AW, Hasilik A, Winchester BG (1998). Application of magnetic chromatography to the isolation of lysosomes from fibroblasts of patients with lysosomal storage disorders. FEBS Lett..

[15] Walker, M. W. & Lloyd–Evans, E. in *Methods in Cell Biology*. Vol. 126 (eds. Platt, F. & Platt, N.) 21–43 (Academic Press, 2015). 10.1016/bs.mcb.2014.10.019 25665439

[16] Tharkeshwar AK (2017). A novel approach to analyze lysosomal dysfunctions through subcellular proteomics and lipidomics: the case of NPC1 deficiency. Sci. Rep..

[17] Zoncu R (2011). mTORC1 Senses Lysosomal Amino Acids Through an Inside-Out Mechanism That Requires the Vacuolar H+-ATPase. Science.

[18] Abu-Remaileh M (2017). Lysosomal metabolomics reveals V-ATPase- and mTOR-dependent regulation of amino acid efflux from lysosomes. Science.

[19] Wyant GA (2018). NUFIP1 is a ribosome receptor for starvation-induced ribophagy. Science.

[20] Vonfigura K, Hasilik A (1986). Lysosomal-enzymes and their receptors. Annu. Rev. Biochem..

[21] Makrypidi G (2012). Mannose 6 Dephosphorylation of Lysosomal Proteins Mediated by Acid Phosphatases Acp2 and Acp5. Mol. Cell. Biol..

[22] Caval T (2019). Targeted Analysis of Lysosomal Directed Proteins and Their Sites of Mannose-6-phosphate Modification. Mol. Cell. Proteom..

[23] Kollmann K (2005). Identification of novel lysosomal matrix proteins by proteome analysis. Proteomics.

[24] Sleat DE (2005). The human brain mannose 6-phosphate glycoproteome: A complex mixture composed of multiple isoforms of many soluble lysosomal proteins. Proteomics.

[25] Sleat DE (2006). Identification and validation of mannose 6-phosphate glycoproteins in human plasma reveal a wide range of lysosomal and non-lysosomal proteins. Mol. Cell. Proteom..

[26] Sleat DE, Della Valle MC, Zheng H, Moore DF, Lobel P (2008). The mannose 6-phosphate glycoprotein proteome. J. Proteome Res..

[27] Sleat DE (2012). Proteomic analysis of mouse models of Niemann-Pick C disease reveals alterations in the steady-state levels of lysosomal proteins within the brain. Proteomics.

[28] Sleat DE (2009). Mass Spectrometry-based Protein Profiling to Determine the Cause of Lysosomal Storage Diseases of Unknown Etiology. Mol. Cell. Proteom..

[29] Qian MQ, Sleat DE, Zheng HY, Moore D, Lobel P (2008). Proteomics analysis of serum from mutant mice reveals lysosomal proteins selectively transported by each of the two mannose 6-phosphate receptors. Mol. Cell. Proteom..

[30] Sleat DE (2013). Extending the Mannose 6-Phosphate Glycoproteome by High Resolution/Accuracy Mass Spectrometry Analysis of Control and Acid Phosphatase 5-Deficient Mice. Mol. Cell. Proteom..

[31] Lowry OH, Rosebrough NJ, Farr AL, Randall RJ (1951). Protein measurement with the Folin phenol reagent. J. Biol. Chem..

[32] Laemmli UK (1970). Cleavage of structural proteins during the assembly of the head of bacteriophage T4. Nature.

[33] Winter D, Steen H (2011). Optimization of cell lysis and protein digestion protocols for the analysis of HeLa S3 cells by LC-MS/MS. Proteomics.

[34] Kollipara L, Zahedi RP (2013). Protein carbamylation: *in vivo* modification or *in vitro* artefact?. Proteomics.

[35] Wiśniewski JR, Zougman A, Nagaraj N, Mann M (2009). Universal sample preparation method for proteome analysis. Nat. Methods.

[36] Muller T, Winter D (2017). Systematic Evaluation of Protein Reduction and Alkylation Reveals Massive Unspecific Side Effects by Iodine-containing Reagents. Mol. Cell. Proteom..

[37] Rappsilber J, Ishihama Y, Mann M (2003). Stop and go extraction tips for matrix-assisted laser desorption/ionization, nanoelectrospray, and LC/MS sample pretreatment in proteomics. Anal. Chem..

[38] Comprehensive proteomic analysis of mouse embryonic fibroblast lysosomes by mass spectrometry (2019). PRIDE.

[39] Ponnaiyan S, Akter F, Singh J, Winter D (2019). figshare.

[40] Foster LJ (2006). A mammalian organelle map by protein correlation profiling. Cell.

[41] Lübke T, Lobel P, Sleat DE (2009). Proteomics of the lysosome. Biochimica et Biophysica Acta (BBA)-Molecular Cell Research.

[42] Mulvey CM (2017). Using hyperLOPIT to perform high-resolution mapping of the spatial proteome. Nature protocols.

[43] Bruderer R, Bernhardt OM, Gandhi T, Reiter L (2016). High-precision iRT prediction in the targeted analysis of data-independent acquisition and its impact on identification and quantitation. Proteomics.

[44] Rosenberger G (2017). Statistical control of peptide and protein error rates in large-scale targeted data-independent acquisition analyses. Nat. Methods.

[45] Chen S, Dong H, Yang S, Guo H (2017). Cathepsins in digestive cancers. Oncotarget.

[46] Biniossek ML, Nagler DK, Becker-Pauly C, Schilling O (2011). Proteomic Identification of Protease Cleavage Sites Characterizes Prime and Non-prime Specificity of Cysteine Cathepsins B, L, and S. J. Proteome Res..

